# Supplementary material for: Gender and Race-Based Health Disparities in COVID-19 Outcomes among Hospitalized Patients in the United States: A Retrospective Analysis of a National Sample
Source: Vaccines (Basel). 2022 Nov 29;10(12):2036. doi: 10.3390/vaccines10122036 (PMC9781042; doi:10.3390/vaccines10122036)
Supplement: Supplementary file 1 [file vaccines-10-02036-s001.zip › vaccines-2020614-supplementary.pdf]

Supplementary Table S1: ICD 10 codes	
**Variable**	**ICD-10 CM code**
STEMI	I21.0X, I21.1X, I21.2X, I21.3X, I21.9X
Covid	U071, U00, U49, U50, U85, J1282
Cardiac arrest	I46.XX, I49.0XX, I97.12XX, I97.71XX
Smoking	F17.XX, Z87.891
CAD	I25.10, I25.11, I25.118, I25.119, I252, I253, I25.4XX, I25.5, I25.6, I25.8XX, I25.7XX, I25.9XX
CHF, HTN, DM, Renal failure, Chronic pulmonary disease, Obesity	Elixhauser comorbidities were used
AKI	N17.XX, N99.0
Stroke	I63.XX, I60.XX, I61.XX, G43.6XX, I97.82XX, I97.81XX
**Variable**	**ICD-10 procedure code**
Intubation	5A1945Z, 5A1955Z, 5A1935Z, 5A09357, 5A09457, 5A09557
Vasopressor use	3E030XZ, 3E033XZ, 3E040XZ, 3E043XZ, 3E050XZ, 3E053XZ, 3E060XZ, 3E063XZ
Hemodialysis	5A1D70Z, 5A1D90Z, 5A1D80Z, 5A1D00Z, 5A1D60Z

Supplemental Table S2. aOR for mortality when compared to White males.

	aOR	95% CI (LL-UL)	P value
White female	0.69	0.67–0.72	<0.001
Black male	0.96	0.92–1.01	0.17
Black female	0.68	0.64–0.71	<0.001
Hispanic male	1.40	1.33–1.47	<0.001
Hispanic female	0.88	0.83–0.93	<0.001
Asian/Pacific islander male	1.27	1.16–1.38	<0.001
Asian/Pacific islander female	0.84	0.76–0.93	<0.001
Native male	1.98	1.67–2.35	<0.001
Native female	1.37	1.14–1.66	<0.001
Other male	1.28	1.18–1.38	<0.001
Other female	0.92	0.84–1.02	0.12

Supplemental Table S3. aOR for intubation when compared to White male.

	aOR	95% CI (LL-UL)	P value
White female	0.65	0.63–0.67	<0.001
Black male	0.94	0.90–0.98	0.007
Black female	0.71	0.68–0.74	<0.001
Hispanic male	1.46	1.39–1.53	<0.001
Hispanic female	0.85	0.81–0.90	<0.001
Asian/Pacific islander male	1.49	1.38–1.61	<0.001
Asian/Pacific islander female	0.90	0.82–0.999	0.04

Native male	1.78	1.53–2.06	<0.001
Native female	1.18	1.01–1.37	0.03
Other male	1.43	1.33–1.53	<0.001
Other female	0.90	0.82–0.988	0.02

Supplemental Table S4. aOR for vasopressor use when compared to White male.

	aOR	95% CI (LL-UL)	P value
White female	0.67	0.62–0.71	<0.001
Black male	1.05	0.93–1.17	0.38
Black female	0.86	0.76–0.97	0.01
Hispanic male	1.67	1.47–1.88	<0.001
Hispanic female	1.01	0.89–1.15	0.78
Asian/Pacific islander male	1.72	1.44–2.06	<0.001
Asian/Pacific islander female	1.16	0.96–1.41	0.11
Native male	1.33	0.83–2.12	0.22
Native female	0.84	0.46–1.54	0.58
Other male	1.46	1.23–1.73	<0.001
Other female	1.08	0.87–1.35	0.43

Supplemental Table S5. aOR for AKI with HD when compared to White male.

	aOR	95% CI (LL-UL)	P value
White female	0.48	0.44–0.52	<0.001
Black male	1.87	1.72–2.05	<0.001
Black female	1.13	1.03–2.13	<0.001
Hispanic male	1.93	1.76–2.13	<0.001
Hispanic female	0.97	0.86–1.08	0.61
Asian/Pacific islander male	1.69	1.44–1.99	<0.001
Asian/Pacific islander female	0.84	0.67–1.07	0.17
Native male	1.88	1.40–2.54	<0.001
Native female	0.81	0.54–1.23	0.34
Other male	1.65	1.42–1.92	<0.001
Other female	0.85	0.68–1.06	0.16

Supplemental Table S6. aOR for AKI when compared to White male.

	aOR	95% CI (LL-UL)	P value
White female	0.60	0.58–0.61	<0.001
Black male	1.92	1.86–2.00	<0.001

Black female	1.08	1.04–1.12	<0.001
Hispanic male	1.10	1.06–1.14	<0.001
Hispanic female	0.62	0.59–0.65	<0.001
Asian/Pacific islander male	1.23	1.15–1.31	<0.001
Asian/Pacific islander female	0.71	0.66–0.77	<0.001
Native male	1.07	0.92–1.25	0.33
Native female	0.63	0.54–0.73	<0.001
Other male	1.23	1.15–1.31	<0.001
Other female	0.73	0.68–0.79	<0.001

Supplemental Table S7. aOR for cardiac arrest when compared to White male.

	aOR	95% CI (LL-UL)	P value
White female	0.63	0.59–0.68	<0.001
Black male	1.43	1.30–1.57	<0.001
Black female	1.05	0.96–1.16	0.24
Hispanic male	1.82	1.65–2.01	<0.001
Hispanic female	1.18	1.06–1.33	0.003
Asian/Pacific islander male	1.78	1.50–2.10	<0.001
Asian/Pacific islander female	0.93	0.76–1.15	0.55
Native male	1.68	1.25–2.26	0.001
Native female	0.90	0.62–1.31	0.60
Other male	1.68	1.44–1.95	<0.001
Other female	1.26	1.06–1.51	0.008

Supplemental Table S8. aOR for stroke when compared to White male.

	aOR	95% CI (LL-UL)	P value
White female	0.83	0.76–0.91	<0.001
Black male	1.31	1.18–1.46	<0.001
Black female	1.04	0.93–1.16	0.45
Hispanic male	1.24	1.11–1.39	<0.001
Hispanic female	0.92	0.81–1.05	0.26
Asian/Pacific islander male	1.34	1.09–1.64	0.005
Asian/Pacific islander female	1.45	1.16–1.81	0.001
Native male	1.17	0.71–1.94	0.52
Native female	0.80	0.50–1.30	0.37
Other male	1.70	1.43–2.03	<0.001
Other female	1.21	0.98–1.51	0.07
